# Antidepressant‐ and Anxiolytic‐Like Effect of the *Froriepia subpinnata* Extract in the Rat: Neurochemical Correlates

**DOI:** 10.1002/brb3.70171

**Published:** 2024-11-28

**Authors:** Mohammad R. Samandari‐Bahraseman, Keyvan Esmaeilzadeh‐Salestani, Manijeh Dogani, Banafsheh Khaleghdoust, Nima Hatami, Saeed Esmaeili‐Mahani, Leila Elyasi, Evelin Loit, Jaanus Harro

**Affiliations:** ^1^ Department of Biology, Faculty of Sciences Shahid Bahonar University of Kerman Kerman Iran; ^2^ Varjavand Kesht Kariman, Limited Liability Company Kerman Iran; ^3^ Chair of Crop Science and Plant Biology, Institute of Agricultural and Environmental Sciences Estonian University of Life Sciences Tartu Estonia; ^4^ Institute of Technology University of Tartu Tartu Estonia; ^5^ Department of Endodontic Dentistry Kerman University of Medical Sciences Kerman Iran; ^6^ Neuroscience Research Center, Department of Anatomy, Faculty of Medicine Golestan University of Medical Science Gorgan Iran; ^7^ Division of Neuropsychopharmacology, Institute of Chemistry University of Tartu Tartu Estonia

**Keywords:** chronic stress, cognition and antidepressant, *F. subpinnata* extract, neurochemistry

## Abstract

**Background:**

The study aims to explore the potential antianxiety effect of *Froriepia subpinnata*, a native plant in northern Iran, and it is considered an antiflatulent, appetizing, antiseptic, antispasmodic, and diuretic. Despite its widespread use in diets and its reputation for calming effects, no prior research has specifically investigated its antianxiety properties.

**Methods:**

Rats were subjected to a variety of stressors for 24 days. Rats were treated with the *F. subpinnata* extract (100, 200, and 400 mg/kg, orally) for 14 days starting from the 10th day of stress. Then behavioral tests (elevated plus‐maze, open field, sucrose preference, Morris water maze, passive avoidance) were examined. Real‐time PCR was used to investigate changes in the expression of candidate genes of stress response and memory. Oxidative stress markers and corticosterone levels in serum were also measured.

**Results:**

Chronic stress reduced performance in a variety of tests of anxiety and memory, and treatment with the *F. subpinnata* extract dose‐dependently improved the behavioral deficits caused by chronic stress. At the dose of 200 mg/kg, the *F. subpinnata* extract mitigated the effect of stress on the expression of several genes, such as those encoding dopamine D_1_ and D_2_ receptors, glutamate NMDA, and AMPA receptor subunits (Grin1 and Gria1, respectively), glucocorticoid and mineralocorticoid receptors, cholecystokinin (CCK) and CCK_B_ receptor, neuropeptide Y, and the GABA_A_ receptor alpha_2_ subunit. Also, the expression of two genes, *TrkB* and *BDNF*, was significantly affected by the extract, demonstrating meaningful decreasing changes. Furthermore, treatment with the extract led to a decrease in oxidative stress and an elevation in cortisol levels in stressed animals.

**Conclusion:**

In this study, we provide the first evidence of the antistress and antianxiety effects of *F. subpinnata* extract, along with its potential procognitive impact on memory.

## Graphical abstract

The potential antianxiety and antidepressant effects of Froriepia subpinnata were explored. Behavioral tests, including the elevated plus‐maze, open field, sucrose preference, Morris water maze, and passive avoidance, were conducted. Real‐time PCR was employed to examine changes in the expression of candidate genes associated with stress response and memory. Oxidative stress markers and corticosterone levels in serum were measured.

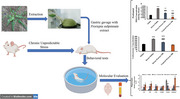

## 1 Introduction

Stress is an integral part of life, which is created by a large variety of physical or psychological stimuli (Hammen 2005). Frequent and unpredictable stressful stimuli can, however, cause conditions such as feeling at a loss of control, restlessness, anxiety, physical ailments, and mental disorders (Spielberger et al. 2014). Environmental physical and psychological stressors can disrupt homeostasis and lead to irreversible cellular damage by either increasing the production of free radicals or reducing their elimination (Srivastava and Kumar 2015). Exposure to chronic stress, followed by increased levels of stress hormones, has various effects on the brain, especially areas such as the hippocampus, amygdala, and prefrontal cortex, which are involved in anxiety and related disorders (Wingenfeld and Wolf 2014). Physical or mental stress increases the activation of the hypothalamic–pituitary–adrenal (HPA) axis (Barden 2004), and activation of this axis increases the levels of corticosterone (Weber, Eckert, and Müller 2006), which in turn affects cognitive functions, including memory, learning, and anxiety through mineralocorticoid (MRs) and glucocorticoid receptors (GRs) (Mantsch and Gasser 2015). A general consequence of stress is the impairment of learning and memory, and it seems that the size of the effect depends on factors such as the type and severity of the stressor, gender, and age (Urban and Valentino 2017).

Stress impacts several neurotransmitter systems in brain regions such as the hippocampus, amygdala, and prefrontal cortex. These systems include the GABAergic, glutamatergic, and dopaminergic pathways (Mora et al. 2012). Notably, stress leads to increased release of gamma‐aminobutyric acid (GABA), the brain's primary inhibitory neurotransmitter, in various regions, including the hippocampus, amygdala, and nucleus accumbens (Sanacora and Saricicek 2007). Stress‐induced release of glutamate, the primary central excitatory neurotransmitter, can lead to anxiety and impair memory and learning. This effect is particularly pronounced through excessive activation of *N*‐methyl‐d‐aspartate (NMDA) type ionotropic receptors (Piri et al. 2020). Similarly, stress‐triggered dopamine release, acting via D_1_ and D_2_ receptors, influence learning processes and memory formation (Bonapersona, Joels, and Sarabdjitsingh 2018). Stress also has a major impact on neurotrophic functions by altering the expression of neuroplasticity‐related proteins, such as the brain‐derived neurotrophic factor (BDNF) (Aydemir et al. 2006).

Current antianxiety drugs often have side effects that restrict their long‐term utility. Recently, researchers have turned their focus toward traditional medicinal plants as potential treatments for brain disorders (Dogani et al. 2021; Khorrami et al. 2023; Rostamabadi, Bahraseman, and Esmaeilzadeh‐Salestani 2023). *Froriepia subpinnata* Ledeb. Bail. (In Persian: Gijavash), a medicinal plant from the *Apiaceae* family, is the only species of the *Froriepia* genera growing naturally in the northern parts of Iran (Darakhshan et al. 2015). In traditional therapies, it is assumed that *F. subpinnata* is soothing and reduces perceived stress (Basma et al. 2011). Previous studies have also shown the antioxidant and anticancer effects of this plant (Azizkhani and Sodanlo 2021; Rostamabadi, Bahraseman, and Esmaeilzadeh‐Salestani 2023). To date, no studies have examined the impact of this plant on stress models, anxiety, and memory. Consequently, the current research aims to investigate the effects of the hydroalcoholic extract of *F. subpinnata* in a chronic stress‐based animal model of depression, focusing on its influence on anxiety, learning, and memory.

## 2 Methods

### 2.1 Aqueous Extract Preparation of *F. subpinnata*

The *F. subpinnata* leaves (edible parts) were collected from Lasht‐e Nesha (37°20ʹ N, 49°51ʹ E), Gilan Province, Iran, on May 15, 2020. Leaves were washed with distilled water and then oven‐dried at 30°C for 3 days. Dried leaves were ground into powder and mixed with methanol (70%). The mixture was shaken for 48 h at room temperature to obtain the extract. To refine and concentrate the extract, the extract was passed through a Whatman filter paper (Grade 1) several times and then placed into a rotary evaporator at 40°C to remove the organic solvent. Voucher specimens were collected, dried by pressing in absorbent paper, stored at room temperature, and lodged at the herbarium of the University of Guilan.

### 2.2 Analyses of Essential Oils Using GC–MS

The GC–MS procedure involves using helium gas with a flow rate of 1 mL/min as the carrier and 250°C as the injector temperature. In the oven, a temperature of 50°C was maintained for 2 min before gradually increasing at a rate of 10°C/min to reach a final temperature of 250°C after 15 min. GC–MS analysis was performed on a trace DSQ GC–MS device manufactured by Thermo Finnigan at 70 eV ionization energy and equipped with the DB‐5 capillary column with helium as the carrier gas. GC–MS system data and literary references were used to determine the relative retention time and mass spectrum of the compounds.

### 2.3 Animals and General Procedure

In this study, 64 male Wistar rats (*Rattus norvegicus*) (180–210 g) were obtained from the Animal Center of Shahid Bahonar University of Kerman and adapted to the laboratory conditions for 2 weeks before starting the experiments (ethics committee approval EC/KNRC/95‐8 A). They were kept in controlled conditions, including standard temperature (25 ± 1°C), humidity (50% ± 10%), and a 12:12‐h light‐dark cycle (lights on at 7:00 a.m.). Then, the animals were randomly assigned to one of the following eight groups (*n* = 8 per group): control, vehicle (saline), extract 200 (200 mg/kg), stress (chronic unpredictable stress), stress + saline, stress + extract 100 (100 mg/kg), stress + extract 200 (200 mg/kg), and stress + extract 400 (400 mg/kg). Saline and extract (dissolved in saline) were administered orally via gastric gavage for 14 consecutive days, starting on the 10th day of the stress (chronic unpredictable stress) procedure. The high‐dose administration of the extract (800, 1600, 3200, and 4000 mg/kg, once a day for 3 days) did not impact the mortality rate in rats. The animals were housed in four rats per cage. Thereafter, behavioral tests were conducted, and animals were euthanized. In this study, animal experiments, clinical trials, and biodiversity rights were considered according to international, national, and institutional legislation.

### 2.4 Chronic Unpredictable Stress

The Paul Willner method (Willner 1984) was applied with some modifications. Several different stressors were used, and they were applied in a variable sequence each week to prevent any habituation. Animals received two different types of stress each day for 24 days. This is explained in detail in Table 1. The experiments were performed based on tail marking of the rats, maintaining a consistent sequence for all tests, randomized across groups.

**TABLE 1:** Protocol used for chronic stress induction for 24 days.

Day	1	2	3	4	5	6	7	8
**Stressor**	15 min forced swim (20°C), 1 min tail pinch	12 h cage tilting (45°C), 1 h cage rotation	Reversal of the light/dark cycle, 1 h cold room (4°C)	12 h wet bedding, crowded cage	24 h food deprivation, 1 h restraint	12 h cage tilting (45°C), crowded cage	24 h water deprivation, 1 h cold room isolation	Reversal of the light/dark cycle, 1 min tail pinch

Day	9	10	11	12	13	14	15	16
**Stressor**	Cold room (4°C), 1 h cage rotation	24 h water and food deprivation, 12 h cage tilting (45°C)	15 min forced swim (20°C), 1 h restraint	Reversal of the light/dark cycle, 24 h food deprivation	1 min tail pinch, cold room (4°C)	24 h water deprivation, 1 h restraint	12 h wet bedding, 12 h cage tilting (45°C)	1 h cage rotation, reversal of the light/dark cycle

Day	17	18	19	20	21	22	23	24
**Stressor**	1 h restraint, crowded cage	12 h wet bedding, 1 min tail pinch	Reversal of the light/dark cycle, 12 h cage tilting (45°C)	15 min forced swim (20°C), 24 h water deprivation	1 h cage rotation, crowded cage	24 h food deprivation, 1 min tail pinch	1 h restraint, 12 h wet bedding	24 h water and food deprivation, crowded cage

*Note*: Restraint (animals were housed in a restrainer made of plexiglass and flexible nylon, which restricted movement but did not affect respiration and air circulation), tail pinch (it means placing the animal in the previously described restrainer and applying a clothespin approximately 1 cm from the base of the tail), crowded cage (eight rats per cage).

### 2.5 Behavioral Tests

#### 2.5.1 Elevated Plus‐Maze Test

In the elevated plus‐maze (EPM) test (Pellow et al. 1985), there were four arms extending from a central platform (10 cm × 10 cm) elevated 50 cm above the ground, two open arms of 50 cm and two enclosed arms of 50 cm each. Light intensity on the maze apparatus was 40–55 lux, and it was located in a quiet room. For 5 min, each animal was allowed to explore the maze facing the open arm on the platform. Video tracking was used to monitor animal behavior in the maze. Animals were observed entering, spending time, and covering the distance in the open arm. Following each test, the surroundings underwent disinfection using 70% alcohol and were subsequently thoroughly dried with a clean cloth. Before the subsequent testing phase, a period was allocated to eliminate any residual alcohol odor from the environment. In accordance with the standard protocol, 70% ethanol (rather than 100% pure ethanol) was employed for disinfection purposes. Following disinfection, the site was meticulously dried using a clean, damp cloth before proceeding to the subsequent test.

It is worth mentioning that the rats were placed in the test environment for habituation 1.5 h before the tests. In addition, the tests were performed between 8:00 a.m. and 6:00 p.m. The intertest intervals varied from 2 h to overnight, depending on the duration of each test.

#### 2.5.2 Open Field Test

The open field test (Hall 1941) was conducted in a wood square arena, measuring 70 × 70 × 30 cm. 5 min were allowed for the animals to explore the field freely. Video cameras were used to record the animals' distance and time spent as well as how often they entered the center of the field to measure anxiety‐like behaviors. The arena was cleaned with ethanol after each animal was tested to eliminate any residual smells.

#### 2.5.3 Sucrose Preference Test

Around 72 h before the test, the rats were housed in cages and given 1% w/v sucrose solution in two bottles. For 24 h, tap water was replaced with 1% sucrose in one bottle. Upon completing the adaptation period, the animals were deprived of food and water for 24 h, and their preference for sucrose was assessed by exposing them for 1 h to two identical bottles filled either with sucrose solution or water. The volume of sucrose consumed versus the total liquid (water + sucrose) consumed during the 1‐h trial was used as a measure of sucrose preference (Willner et al. 1987).

#### 2.5.4 Morris Water Maze

To conduct the Morris water maze test (Morris 1981), a black circular tank, 150 cm in diameter and 75‐cm high, was filled with tap water, and its temperature was set at 21°C. Platforms made of Plexiglas were placed 1 cm below the water level. The black tank was located inside a room with several visual cues surrounding the circular pool. Video cameras were installed on top of the tank to record the animals' swimming behavior. During the training phase, the distance and duration of the search for the hidden platform were measured, along with the number of entries, the duration of the time spent in the target quadrant, and the distance traveled (during the retention phase). Learning trials (training phase) consisted of four blocks (four trials per block) with a 20‐min rest between each block. Each of the four randomly selected directions was used to begin the trial. All animals were permitted to swim for 1 min and find a concealed platform placed in the middle of a fixed quadrant. Whenever the rat failed to find the hidden platform within 1 min, it was removed from the water and gently guided to the platform, where it remained for 30 s between trials. Finally, 24 h after the last block, the platform was retrieved from the pool, and the animals were allowed to look for it for 1 min on test day (retention phase).

#### 2.5.5 Passive Avoidance Learning

There were two chambers, one for light and one for dark, with a grid‐like floor (Ader, Weijnen, and Moleman 1972). The chambers were connected by a polymethyl methacrylate (Plexiglass) gate. Thirty minutes before the acquisition trial, the rats were placed in the light chamber, allowed to enter the dark chamber at their leisure, and were habituated to it. During the acquisition phase, rats were placed in the light chamber individually, and the guillotine gate opened after 5 s. The guillotine door was lowered upon entering the dark chamber, and an electric shock was delivered once through the grid floor (0.5 mA, 50 Hz, 4 s).

Rats were returned to their home cage after 30 s, and 5 min later the same procedure was repeated. A successful passive avoidance learning procedure was defined as animals not entering the dark room for 300 s. Otherwise, the rats received the shock again. The number of acquisition sessions was recorded.

After 24 h, the rats were placed in the light chamber and allowed to cross into the dark chamber. Step‐through latency was recorded up to a maximum of 5 min.

### 2.6 Biochemical Assays

#### 2.6.1 Tissue and Serum Sampling

Following the treatment period, the animals were anesthetized with ether to assess the impact of the extract on cortisol hormone levels. Subsequently, blood was collected via cardiac puncture, and animals were decapitated. The serum was obtained by centrifuging the blood at 0.6 × *g* for 15 min at 4°C. Finally, hippocampal and prefrontal cortex tissues were extracted from the animals.

#### 2.6.2 Malondialdehyde Assay

Malondialdehyde (MDA) is a common by‐product of lipid peroxidation during oxidative stress (Heath and Packer 1968). 0.1 g of tissues were homogenized in 0.1% trichloroacetic acid (TCA), followed by centrifugation at 9300 × *g* for 15 min. Supernatant 1.0 mL was treated with 4.0 mL of 0.5% thiobarbituric acid in 20% TCA. Heat the mixture at 95°C for 30 min and then cool it in an ice bath. The supernatant was centrifuged for 10 min, and the absorbance at 532 nm was measured (Heath and Packer 1968).

#### 2.6.3 H_2_O_2_ Assay

The levels of H_2_O_2_ in the tissues were measured according to Velikova et al. (2000). 0.1 g of tissues were homogenized in 1 mL of TCA (pH 7.4). Homogenate was centrifuged for 10 min at 4°C at 10,000 × *g*. To measure H_2_O_2_, 1 mL of potassium iodide 1 mM and 0.5 mL of phosphate buffer (10 mM; pH 7.4) were added to the tissue supernatant. A spectrophotometric measurement at 390 nm was carried out to determine the level of H_2_O_2_.

#### 2.6.4 Corticosterone Assay

The sera were aliquoted and stored at 80°C until assays were performed. A rat‐specific ELISA kit (ZellBio GmbH, Ulm, Germany) was used to measure corticosterone levels in serum. The sensitivity of the corticosterone assay was 1.63 ng/L. Corticosterone's coefficients of variability were 4.7% intra‐assay and 6.3% inter‐assay.

### 2.7 Gene Expression Evaluation

#### 2.7.1 RNA Extraction and Reverse Transcription

We euthanized all animals 24 h after the last behavioral test. The hippocampal and prefrontal cortical tissue was quickly dissected and frozen on dry ice and then stored at −80°C until RNA extraction. Afterward, total RNA was extracted (five randomly chosen rats in each group) by the Trizol reagent (Zaver Zist Azma, Iran). A reverse transcriptase enzyme called M‐MuLV was used for cDNA synthesis (Solis BioDyne, Germany) according to the manufacturer's instructions.

#### 2.7.2 Quantitative Polymerase Chain Reaction

Quantitative RT‐PCR was carried out using real‐time PCR with the SYBR‐Green reporter dye. cDNA prepared from each sample was applied to quantify the mRNA expression of genes encoding BDNF, tropomyosin receptor kinase B (*TrkB*), *Gr*, *Mr*, dopamine D_1_ and D_2_ receptors, *Gaba_a_* receptor alpha‐2 subunit (*Gabra2*), neuropeptide Y (*Npy*), cholecystokinin (*Cck*), CCK_B_ receptor (*Cckbr*), glutamate AMPA‐type receptor subunit gene *Gria1* and NMDA‐type receptor subunit gene *Grin1*, using a Qiagen detection system (Qiagen, Germany). Glyceraldehyde 3‐phosphate dehydrogenase (*Gapdh*) was used as an internal control for normalizing the expression of target genes. The Real Q Plus 2x Master Mix (Solis BioDyne) was applied in the PCR reactions to amplify the cDNA sequences. A list of primer sequences, RT‐PCR product lengths, and NCBI accession numbers is provided in Table S1. A duplicate evaluation of each sample was conducted, and the means were used for analysis. Standard curves generated by increasing the amount of cDNA were used to determine the linearity and efficiency of PCR amplification. The relative mRNA level was calculated using the expression $2^{-\Delta \Delta C_{T}}$.

### 2.8 Statistical Analysis

SPSS 19.0 was used to analyze the data. All results were expressed as mean ± SEM. To determine whether there were statistically significant differences between groups, a one‐way analysis of variance was conducted, followed by a Tukey post hoc test. In addition, before the primary analyses were conducted, data normality was assessed using Prism software. Specifically, the Anderson–Darling test, Shapiro–Wilk test, and Kolmogorov–Smirnov test were employed to evaluate the distribution's normality.

## 3 Results

### 3.1 GC/MS Analysis

**FIGURE 1:**
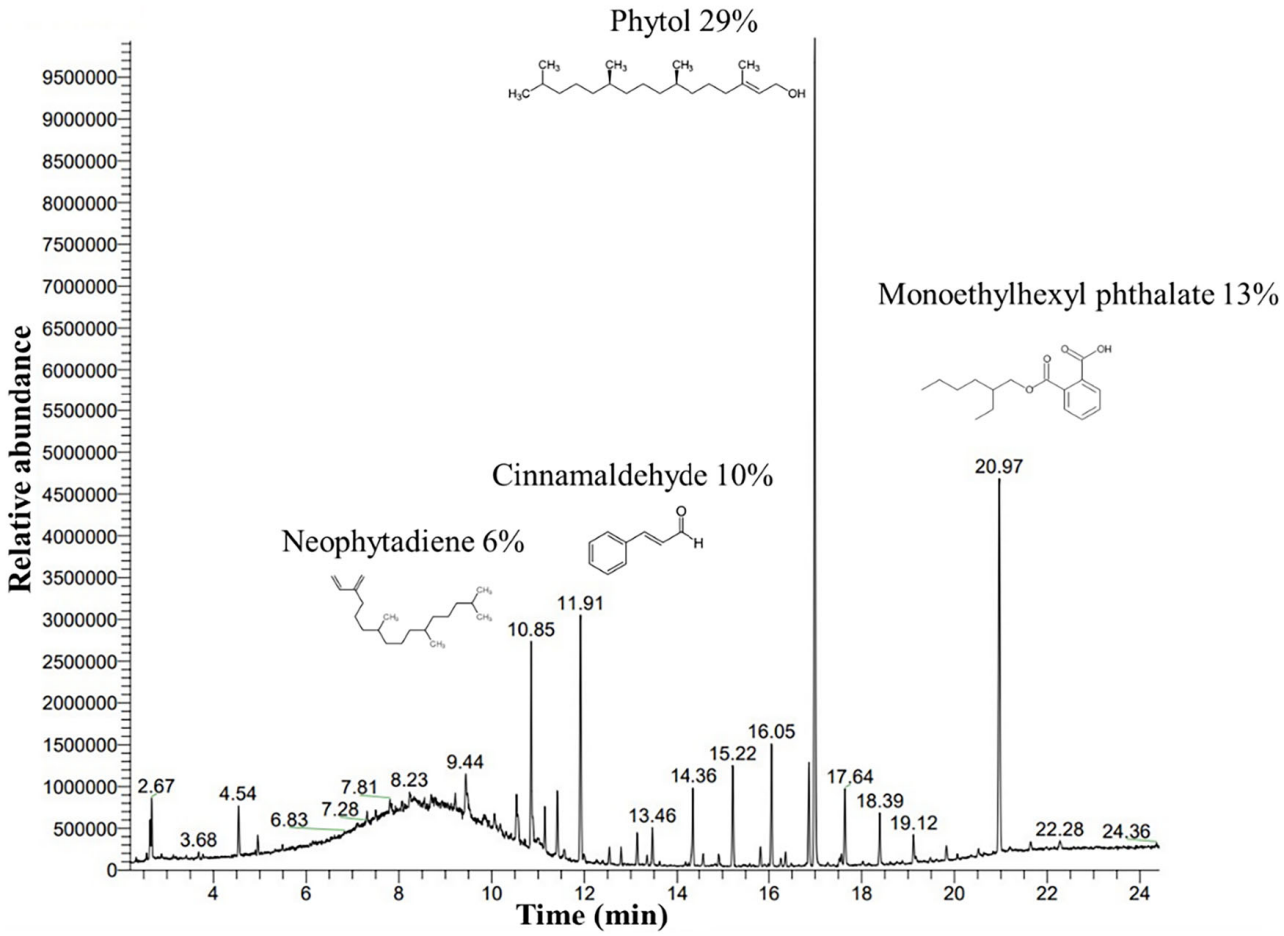
The main compound identified in *Froriepia subpinnata* by GC/MS.

To identify bioactive compounds contained in *F. subpinnata* leaf extract, GC/MS analysis was performed. *F. subpinnata* was found to contain 30 compounds, as determined by the GC/MS chromatogram. This plant contained the most significant concentrations of phytol, monoethylhexylphthalate, cinnamaldehyde, and neophytadiene (NPT) (Figure 1). There were also other compounds identified, each one accounting for less than 2% of the *F. subpinnata* essential oil (Rostamabadi, Bahraseman, and Esmaeilzadeh‐Salestani 2023).

### 3.2 Effect of the *F. subpinnata* Extract on Behavior

#### 3.2.1 EPM Test

**FIGURE 2:**
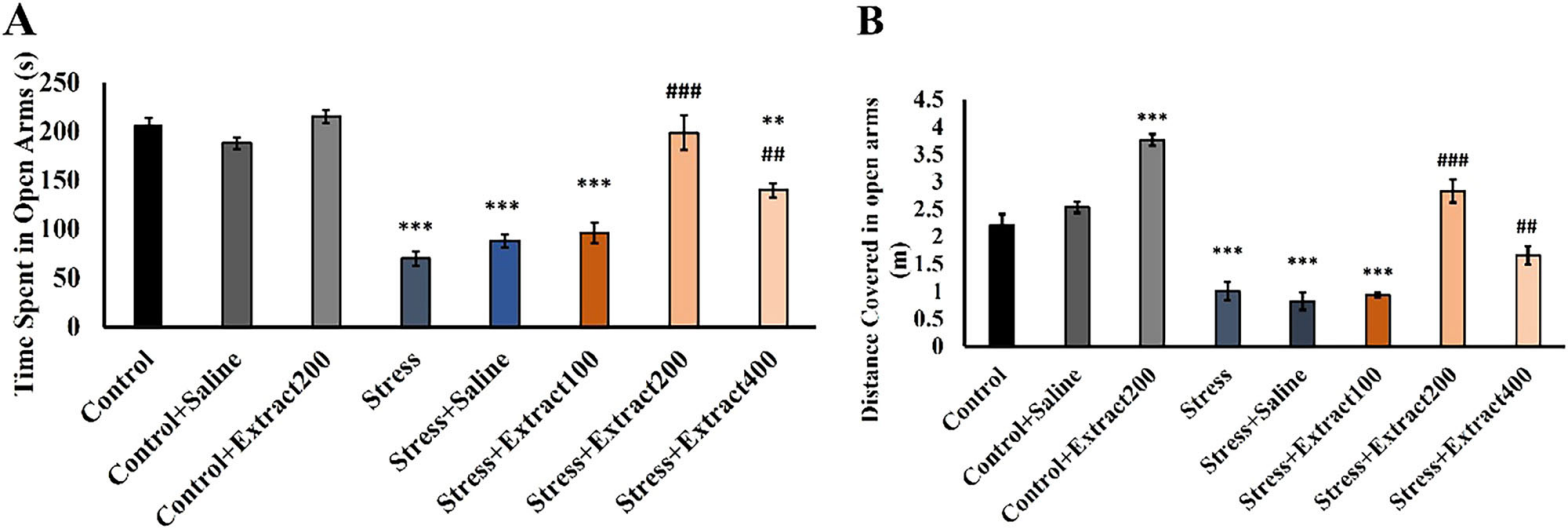
Effect of chronic unpredictable stress and treatment with the *Froriepia subpinnata* extract on (A) time spent in the open arms and (B) distance covered in the open arms. Data are expressed as mean ± SEM. *p < 0.05, **p < 0.01, and ***p < 0.001 compared to control group; ^#^ *p* < 0.05 and ^##^ *p* < 0.01 versus the stress group; ^^^^ *p* < 0.01 and ^^^^^ *p* < 0.001 versus the stress + extract 100 group; ^+^ *p* < 0.05 and ^++^ *p* < 0.001 versus the stress + extract 200 group. Number of tested rats for each group = 8.

Chronic mild stress significantly decreased the time spent (*F*(7, 54) = 12.02, *p *< 0.001; Figure 2A) and distance covered by the rats in the open arms (*F*(7, 54) = 37.77, *p *< 0.001; Figure 2B) when compared to the control group (*p *< 0.001 and *p* < 0.001, respectively). At the doses of 200 and 400 mg/kg, subchronic administration of the *F. subpinnata* extract reversed the effect of stress. At the dose of 200 mg/kg, the *F. subpinnata* extract significantly increased the number of entries into the open arms (*F*(7, 54) = 1.06, *p *< 0.05) in both control and stress conditions (*p *< 0.01 and *p *< 0.001, respectively) (Figure S2). The stress + extract 200 group exhibited the most effective reduction in anxiety behavior compared to other extract groups.

#### 3.2.2 Open Field Test

**FIGURE 3:**
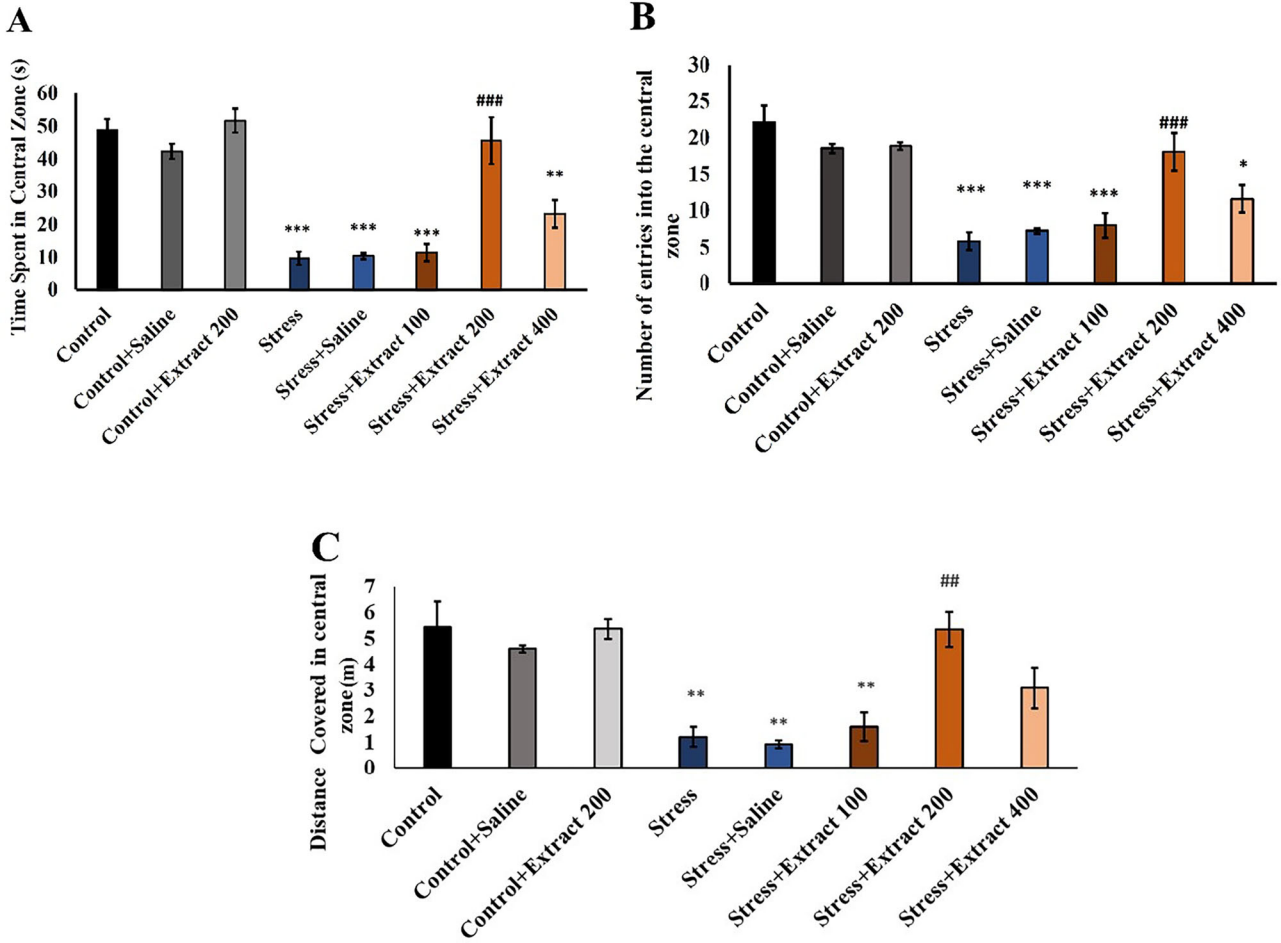
Effect of chronic unpredictable stress and treatment with the *Froriepia subpinnata* extract on open field behavior. (A) Time spent in the central zone. (B) Number of entries into the central zone. (C) Distance covered in the central zone. Data are expressed as mean ± SEM. *p < 0.05, **p < 0.01, and ***p < 0.001 compared to the control group and ^###^ *p* < 0.001 versus the stress group; ^^^^ *p* < 0.01 versus the stress + extract 100 group; ^+^ *p* < 0.05 versus the stress + extract 200 group. Stress: chronic unpredictable stress. Number of tested rats for each group = 8.

Chronic stress reduced the number of entries (*F*(7, 54) = 16.36, *p *< 0.001) and the amount of time spent in the central zone (*F*(7, 54) = 32.11, *p *< 0.001), as well as the distance covered (*F*(7, 54) = 8.37, *p *< 0.001) in the open field (Figure 3A–C). *F. subpinnata* extract at the dose of 200 mg/kg enhanced the number of entries into the central zone (*p* < 0.001), the time spent, and the distance covered in that area (*p* < 0.001 and *p* < 0.01, respectively) in comparison to that behavior in the stress group. Also, there was no significant difference in the mentioned behavioral parameters in the stress + extract 400 and stress + extract 100 compared to the stress group. In comparison to other extract groups, the stress + extract 200 demonstrated the most effective reduction in anxiety behavior.

#### 3.2.3 Morris Water Maze

**FIGURE 4:**
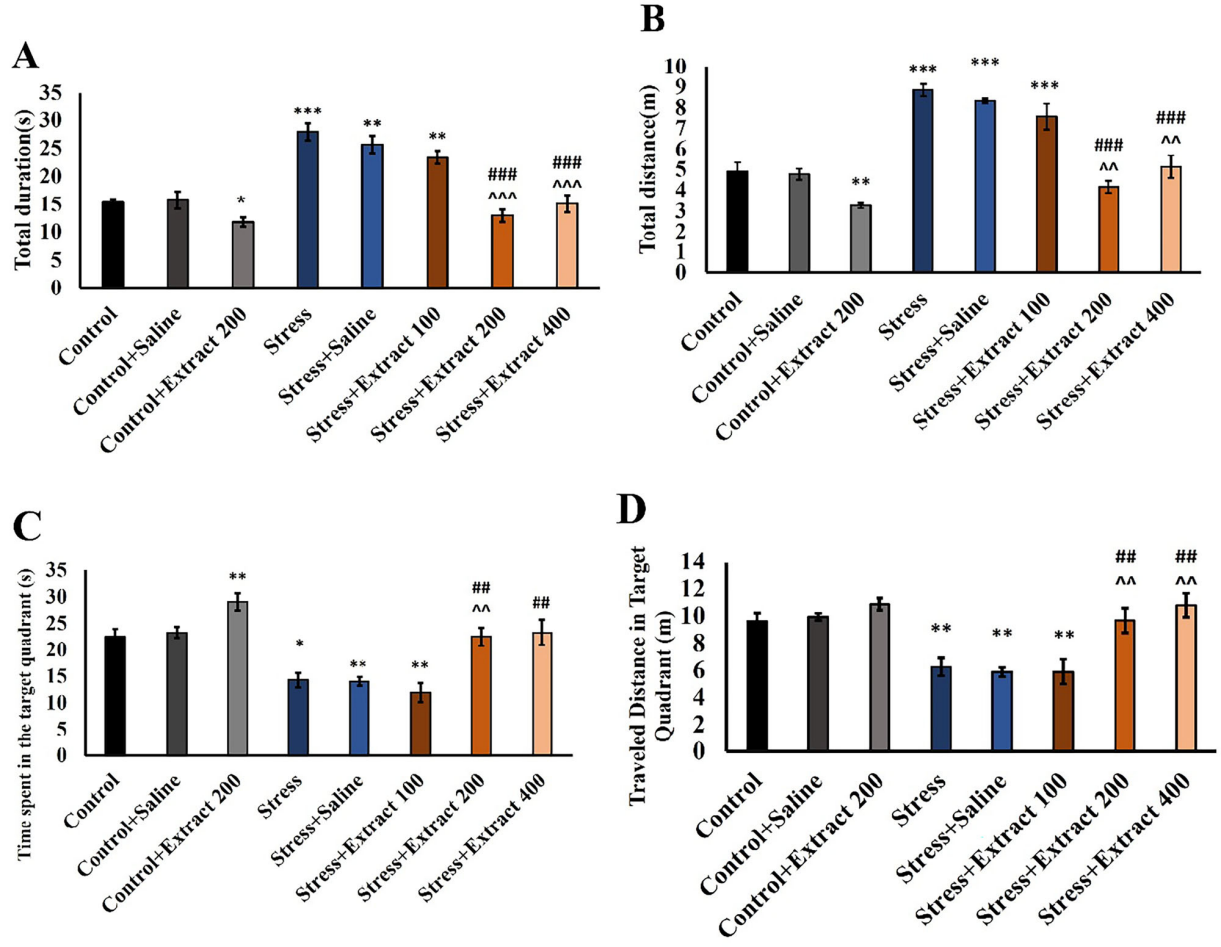
Effect of chronic unpredictable stress and treatment with the *Froriepia subpinnata* extract on spatial learning and memory in animals. (A) Total duration to find the hidden platform in the training phase. (B) Total distance to find the hidden platform during the training phase. (C) Time spent in the target quadrant during the test. (D) Traveled distance in the target quadrant during the test. Data are expressed as mean ± SEM. *p < 0.05, **p < 0.01, and ***p < 0.001 compared to the control group; ^#^ *p* < 0.05 and ^##^ *p* < 0.01 versus the stress group, ^^^ *p* < 0.05, ^^^^ *p* < 0.01 and, ^^^^^ *p* < 0.001 versus the stress + extract 100 group. Number of tested rats for each group = 8.

The total duration (*F*(7, 54) = 26.02, *p *< 0.001) and total traveled distance (*F*(7, 54) = 29.26, *p *< 0.001) to find the hidden platform (Figure 4A,B) in the training phase were significantly affected (*p* < 0.001 for both). Chronic stress increased these measures (*p* < 0.001). Administration of the *F. subpinnata* extract at the two higher doses mitigated this effect of stress. The dose of 200 mg/kg had a small but statistically significant (*p* < 0.001) effect also in control animals. Two higher doses demonstrated a substantial improvement in learning test performance compared to the stress + extract 100 group.

After the learning phase, chronic stress reduced the time spent (*F*(7, 54) = 15.71, *p *< 0.001) and the distance traveled (*F*(7, 54) = 10.90, *p *< 0.001) at the area of the platform (Figure 4C,D). Similarly, to the training phase, administration of the *F. subpinnata* extract reversed the effect of stress. Two higher doses exhibited significant enhancement in memory test performance when compared to the stress + extract 100 group.

#### 3.2.4 Passive Avoidance Learning Test

**FIGURE 5:**
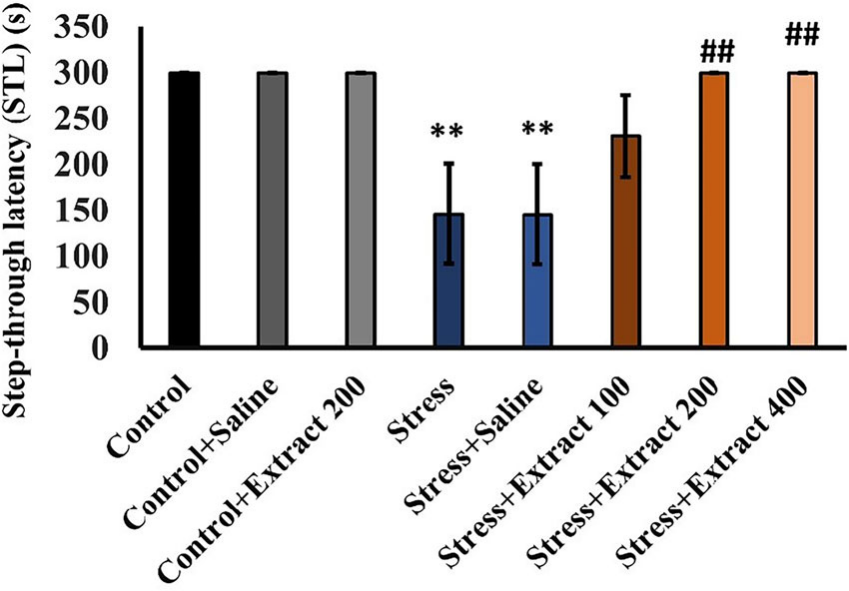
Effect of chronic unpredictable stress (stress) and treatment with the *Froriepia subpinnata* extract on passive avoidance learning in animals. Step‐through latency (STL). Data are expressed as mean ± SEM. **p < 0.01 is a significant level compared to the control group. ^##^ *p* < 0.01 is a significant level versus the stress group. Number of tested rats for each group = 8.

As indicated in Figure S3, in all groups, the number of acquisitions (*F*(7, 54) = 1.13, *p *< 0.35) in the learning test was not significantly different between groups. While control rats did not enter the dark chamber after learning that chronic stress decreased the latency time (*F*(7, 54) = 4.66, *p *< 0.001) to entry (Figure 5). Administration of the *F. subpinnata* extract counteracted this effect of stress. At the doses of 200 and 400 mg/kg, treatment with the extract virtually prevented entry into the dark chamber.

#### 3.2.5 Sucrose Preference Test

**FIGURE 6:**
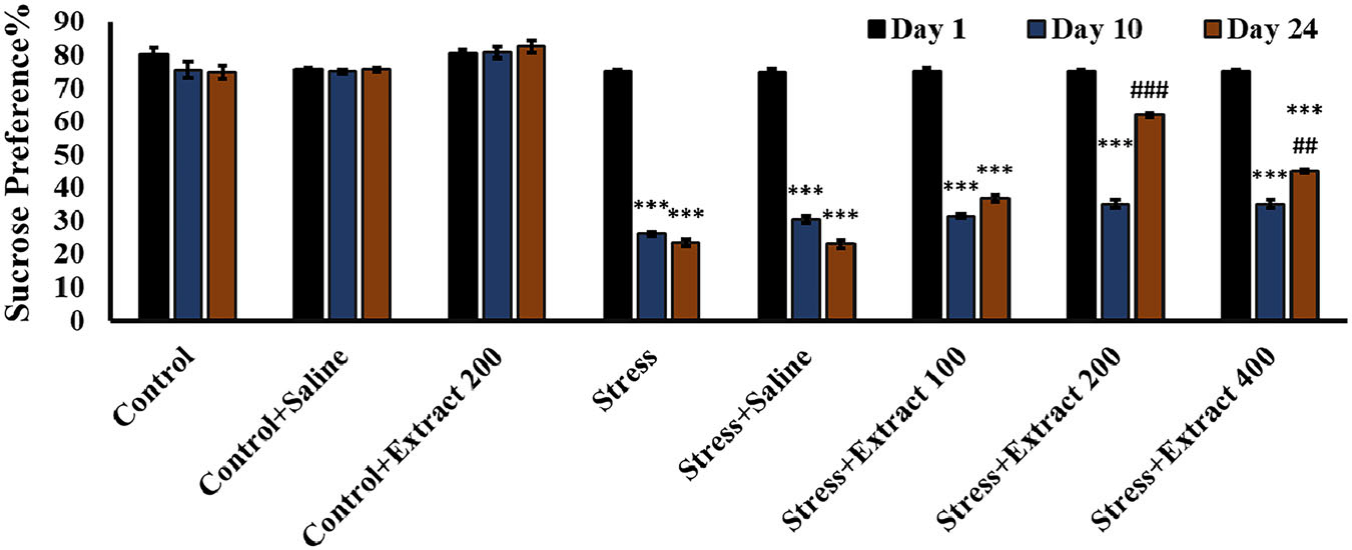
Effect of chronic unpredictable stress (stress) and treatment with the *Froriepia subpinnata* extract on sucrose preference. Data are expressed as mean ± SEM. ***p < 0.001 and ^###^ *p* < 0.01 are significant levels compared to Days 1 and 10, respectively. Number of tested rats for each group = 8.

After 10 days of chronic stress, sucrose preference was significantly decreased (Figure 6). After starting the treatment with the *F. subpinnata* extract from the 10th day (*F*(7, 54) = 1978, *p *< 0.001) onwards, the sucrose preference of the stressed animals increased by the 24th day (*F*(7, 54) = 2647, *p *< 0.001) almost to the level of control at the dose of 200 mg/kg and somewhat less at the dose of 400 mg/kg. The extract did not affect control animals.

### 3.3 Effect of the *F. subpinnata* Extract on Corticosterone Levels in Plasma

**FIGURE 7:**
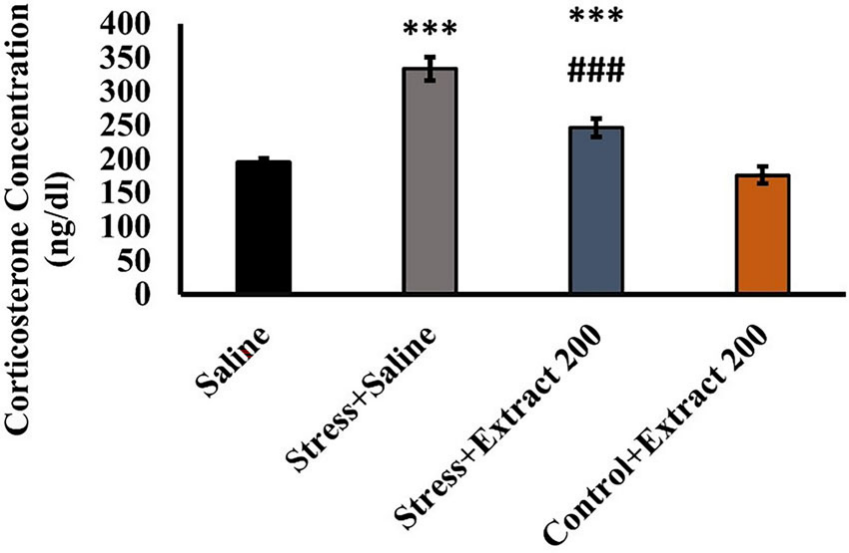
Effect of chronic unpredictable stress (stress) and treatment with the *Froriepia subpinnata* extract (200 mg/kg) on corticosterone levels in serum. Data are expressed as mean ± SEM. ***p < 0.001 compared saline; ^###^ *p* < 0.01 versus stress + saline. Number of tested rats for each group = 4.

As shown in Figure 7, corticosterone levels (*F*(3, 23) = 222.2, *p *< 0.001) were higher in animals exposed to chronic unpredictable stress. *F. subpinnata* extract administration (200 mg/kg) caused a significant decrease in corticosterone levels in stressed animals.

### 3.4 Effect of the *F. subpinnata* Extract on MDA and H_2_O_2_ Levels

**FIGURE 8:**
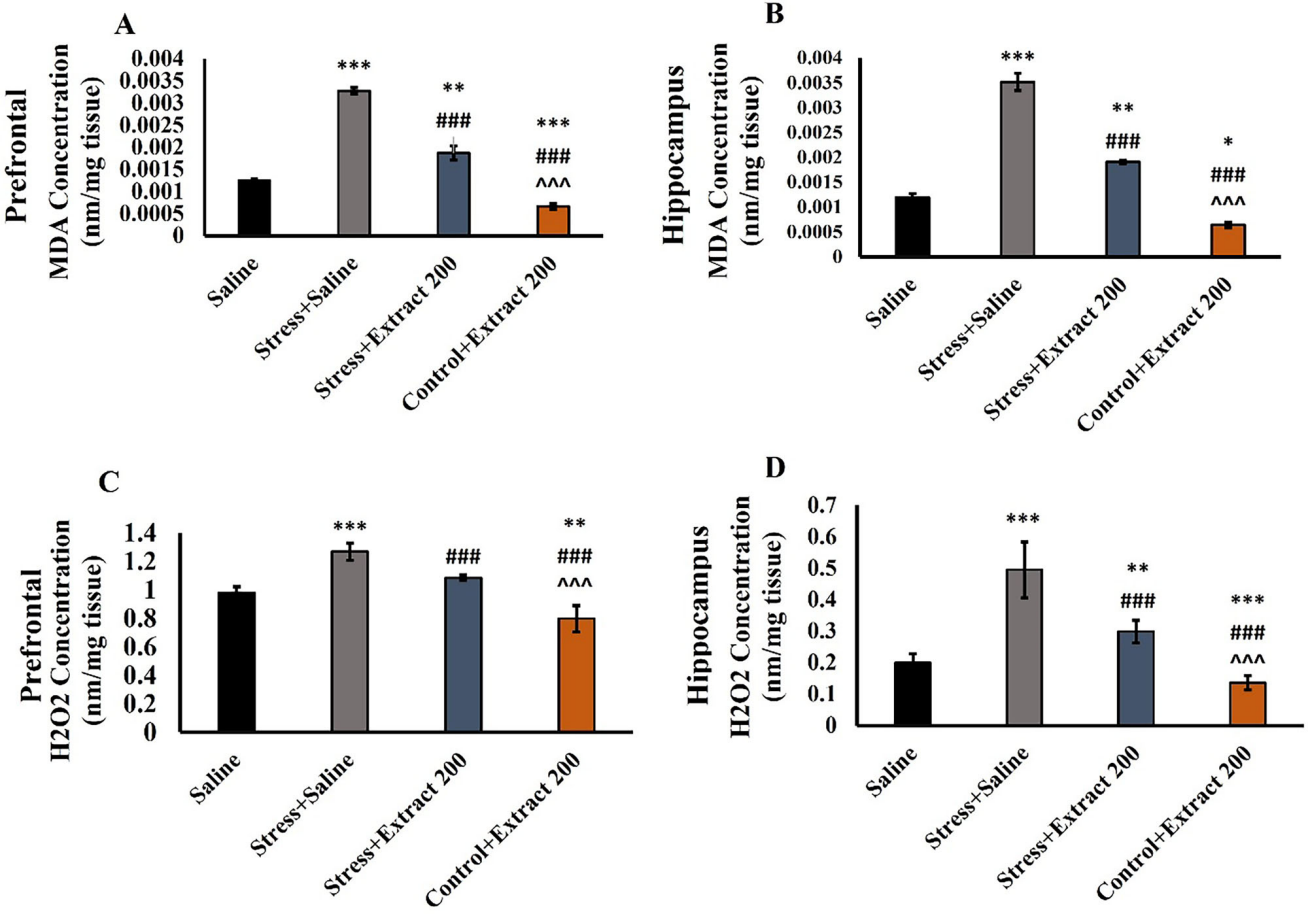
Effect of chronic unpredictable stress (stress) and treatment with the *Froriepia subpinnata* (200 mg/kg) on malondialdehyde (MDA) (A, B) and H_2_O_2_ (C, D) levels. Data are expressed as mean ± SEM. *p < 0.05, **p < 0.01, and ***p < 0.001 to compared saline; ^###^ *p* < 0.01 versus stress + saline; ^^^^^ *p* < 0.01 versus stress + extract. Number of tested rats for each group = 4.

Figure 8A,B shows that chronic stress resulted in a marked increase in the levels of the hippocampal (*F*(3, 23) = 280.4, *p *< 0.001) and prefrontal MDA (*F*(3, 23) = 262, *p *< 0.001). The *F. subpinnata* extract (200 mg/kg) itself reduced the level of MDA significantly (*p* < 0.001 for both), and it also reduced the levels of MDA increased by stress.

The results of the H_2_O_2_ levels were largely similar (Figure 8C,D) in that chronic stress caused a significant rise, but treatment with the *F. subpinnata* extract (200 mg/kg) significantly reduced H_2_O_2_ levels in both prefrontal (*F*(3, 23) = 81.10, *p *< 0.001) and hippocampal (*F*(3, 23) = 69.26, *p *< 0.001) tissue. A statistically significant reduction was also observed after the *F. subpinnata* extract was administered to control animals.

### 3.5 Effect of the *F. subpinnata* Extract on the Expression of Selected Genes Involved in the Regulation of Cognition and Affect

Chronic stress caused a significant decrease in the expression of *Bdnf*, *TrkB (Ntrk2)*, *Gr*, *Mr*, *Gabra2*, *Npy*, *Cck*, and *Cck‐Br* and an increase in the expression of genes encoding dopamine D_1_ and D_2_ receptors and AMPA (*Gria1*) in both the frontal cortex and hippocampus (Figure 9). Treatment with the *F. subpinnata* extract (200 mg/kg) mostly eliminated these effects of stress. The *F. subpinnata* extract (200 mg/kg) treatment had a few effects on its own (it increased expression of the *Npy* and *Gabra2* genes and the *Bdnf, Mr*, and *Gr* genes in the prefrontal cortex).

FIGURE 9Effect of chronic unpredictable stress (stress) and treatment with the *Froriepia subpinnata* extract (200 mg/kg, po.) on gene expression relative to the saline group. (A, C) Hippocampus (B, D) prefrontal. Data are expressed as mean ± SEM. *p < 0.05, **p < 0.01, and ***p < 0.001 compared to the control group; ^###^ *p* < 0.01 versus stress + saline. Number of tested rats for each group = 4.

**Figure:**
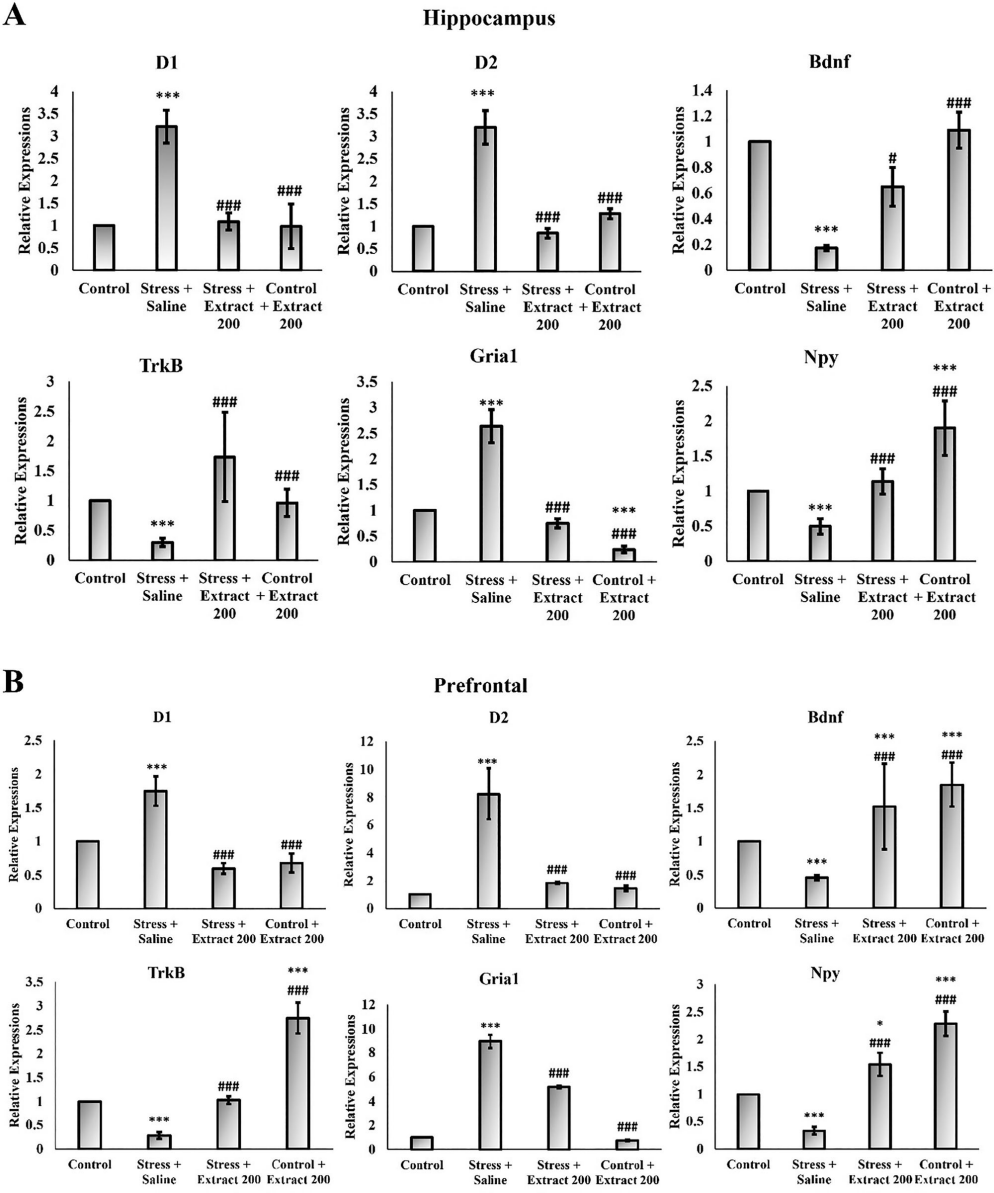

**Figure:**
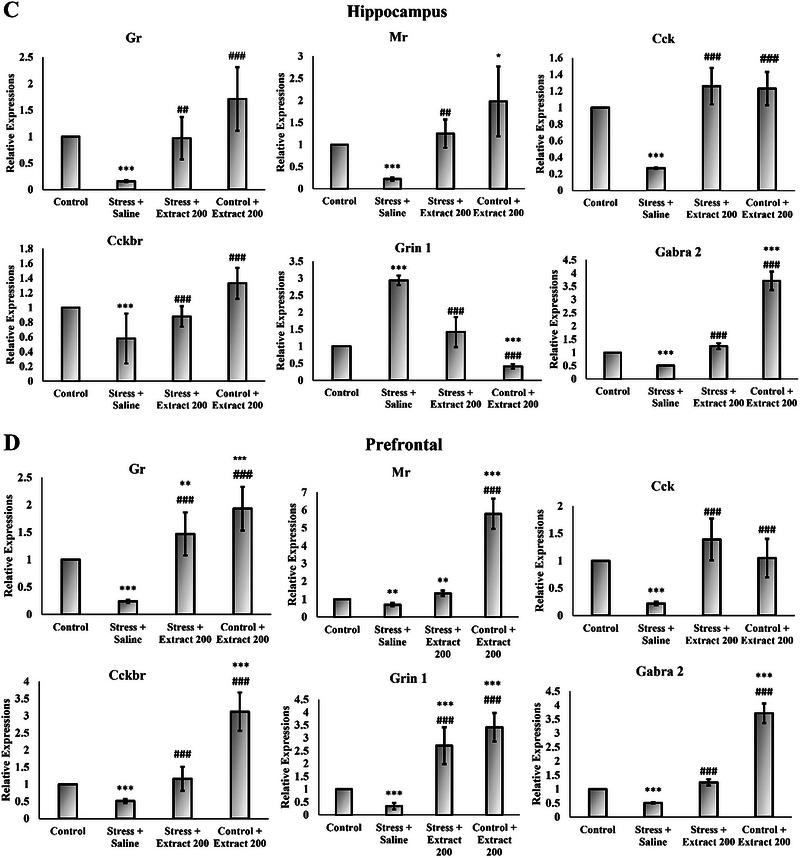

Among all genes, the NMDA (*Grin1*) showed a completely different expression pattern between the hippocampus and frontal cortex. While the NMDA (*Grin1*) decreased under the influence of stress in the prefrontal tissue, and this effect was eliminated under the treatment of the extract, its expression level increased in the hippocampus tissue under stress treatment, and the extract treatment reduced this increase in expression.

## 4 Discussion

The objective of this study was to explore the potential protective effects of the hydroalcoholic extract of *F. subpinnata* against chronic unpredictable stress, focusing on anxiety‐like behavior, learning, memory, and related molecular mechanisms. As expected, chronic unpredictable stress led to increased anxiety‐like behavior and impaired learning and memory. However, treatment with *F. subpinnata* extract demonstrated a dose‐dependent reduction in anxiety‐like behavior and alleviation of memory and learning deficits induced by stress. In summary, the 200 mg/kg extract demonstrated superior performance in mitigating the effects of chronic unpredictable stress. Notably, the control + extract 200 group exhibited significant improvements in memory and learning, along with reduced anxiety‐like behavior compared to the control group. It is worth noting that the lack of a drug control group was one of the limitations of the current experiment.

In traditional medicine, *F. subpinnata* is utilized as a sedative (Seyedabadi et al. 2016). Previous research has demonstrated that *F. subpinnata* possesses antioxidant, antimicrobial, and antibacterial properties (Mohammadzadeh et al. 2018). These beneficial effects are attributed to natural compounds found in the plant, including phytosterols, phenolic acids, flavonoids, thymol, and phytol (Bahrami, Jamzad, and Sedaghat 2021). Notably, phytol, a diterpene alcohol known for its antioxidant properties (Kim et al. 2022), may contribute to the observed sedative and stress‐protective effects of *F. subpinnata* extract, possibly through interactions with GABAergic, monoaminergic systems, and BDNF pathways (Rodríguez‐Landa et al. 2022).

Unpredictable chronic stress increased serum corticosterone levels and thus significantly affected the HPA axis, which was expected (Mousavi, Askari, and Vaez‐Mahdavi 2020). *F. subpinnata* extract also counteracted the effect of stress on GR and MR receptor expression.

Many key genes for stress‐related neurochemistries were found to be impacted by stress, while the stress effects were mitigated or alleviated by the *F. subpinnata* extract. Glucocorticoids play an important role in modulating GABA_A_ receptors (Majewska 1987), and a variety of stressors have been shown to affect GABAergic transmission in the brain (Levy and Tasker 2012). *F. subpinnata* extract contains substantial amounts of monoterpene hydrocarbons such as β‐mirsen, α‐apinene, and limonene (Mirzania, Sarrafi, and Moridi Farimani 2019), and it has been reported that terpenes act on the GABA system. For example, limonene exerts its antianxiety and antistress effects by increasing GABA (but also serotonin levels) in the brain and inhibiting the HPA axis (Zhou, Yoshioka, and Yokogoshi 2009). Phytol has the potential to interact with GABA receptor subunits, which play a role in the anxiolytic effects of diazepam. Anxiolytics enhance GABAergic neuroinhibition by opening chloride channels. The sedative and anxiolytic properties of phytol arise from its interactions with GABA_A_ receptors, specifically the receptor subtypes associated with benzodiazepine effects (Costa et al. 2014; Javaid et al. 2021). In the present study, *F. subpinnata* extract substantially increased the expression of the gene encoding the GABA_A_ receptor alpha‐2 subunit. Thus, some of the compounds in *F. subpinnata* are likely to elicit antianxiety and antistress effects by modulating GABAergic inhibition.

By increasing the effect of glucocorticoid hormones and the activity of glutamate as the main excitatory neurotransmitter, chronic stress may impair learning and memory (Wong et al. 2007). In the present study, stress markedly affected the expression of subunits of both AMPA (*Gria1*) and NMDA (*Grin1*) receptors, albeit the latter was regulated in opposite directions in the hippocampus and cortex. *F. subpinnata* always had an effect opposite to chronic stress and could reduce the stress effects that possibly provide a mechanism for its procognitive action.

BDNF is directly involved in neural growth and regulates survival, differentiation, and maintenance of function in different neural populations. Previous studies support the role of BDNF in regulating the response to stress and behavioral disorders. BDNF expression changes in response to stress, indicating a persistent functional impairment in BDNF regulation (Suri et al. 2013). Animal studies in vivo and in vitro have shown that BDNF levels in limbic structures and serum increase in response to chronic antidepressant use (Mohseni‐Moghaddam et al. 2022). Frank et al. found that injection of BDNF into the midbrain had antidepressant effects (Frank et al. 1997). NPT, a diterpene present in *F. subpinnata*, demonstrates anxiolytic, anticonvulsant, and antidepressant‐like properties. Molecular docking studies suggest that NPT interacts with the GABAergic system, potentially exerting anxiolytic effects without affecting motor coordination or causing sedation (Bhardwaj et al. 2020; Gonzalez‐Rivera et al. 2023).

Multiple neuropeptides are co‐released with classic neurotransmitters to fine‐tune neurotransmission (Hökfelt et al. 2018), and two of the most prominently involved in anxiety regulation are neuropeptide Y (NPY) and cholecystokinin (CCK) (Harro 2006). A unique property of NPY is its ability to potently relieve stress, reduce anxiety, and protect against brain damage (Kask, Rägo, and Harro 1998; Kask et al. 2002). Stress exposure alters the biosynthesis of NPY in distinct brain regions (Serova et al. 2013). Antidepressant drugs elicit an increase in NPY levels in the brain (Heilig, Wahlestedt, and Widerlöv 1988). The upregulation of *Npy* expression in the hippocampus and prefrontal cortex following treatment with *F. subpinnata* extract may play a role in the robust anxiolytic effect observed in our study.

Cholecystokinin (CCK) is widely expressed in the cortex and hippocampus, where it influences the function of memory (Sadeghi, Radahmadi, and Reisi 2015). CCK has also been implicated in anxiety (Harro, Vasar, and Bradwejn 1993) and anxious temperament, and CCK_B_ receptor regulation is altered in anxious states in animals and humans (Harro, Lang, and Vasar 1990, Harro, Marcusson, and Oreland 1992). CCK has significant modulatory effects on the release of dopamine, GABA, and serotonin (Hökfelt et al. 2002; Yaksh et al. 1987). It also affects the expression of BDNF. Social stress triggers the release of CCK in the prefrontal cortex. Interaction of CCK with CCKB receptors in this brain region is crucial for the development of behavioral deficits associated with social defeat (Vialou et al. 2014). Stress has a profound effect on cognition, and CCK probably acts as a mediator for its action (Mitchell et al. 2011). Our findings suggest that elevated concentrations of *Cck*, during stress could potentially mitigate the impact of stress on the brain. Notably, in a study involving chronically stressed rats, systemic CCK administration counteracted memory impairment by inhibiting the HPA axis. Consequently, CCK has been proposed as a central modulator in stressful contexts, and long‐term low‐dose CCK treatment may alleviate memory deficits associated with chronic stress (Mitchell et al. 2011). Furthermore, endogenous CCK release can mediate a safety signal (Wiertelak, Maier, and Watkins 1992). The extract of *F. subpinnata* mitigates stress effects by upregulating the expression of *Cck* and its predominant brain receptor subtype. Speculatively, CCK administration may enhance long‐term potentiation in the CA1 hippocampal region by reducing K^+^ conduction in CA1 pyramidal cells via CCK_B_ receptors. Additionally, CCK induces Ca^2+^ signaling through extracellular calcium influx, leading to NMDA receptor inhibition. Furthermore, increased BDNF expression, possibly mediated by CCK, could contribute to stress protection (Hwang, Kim, and Chun 2013). Overall, CCK's indirect modulation of the HPA axis may safeguard the hippocampus from stress‐related damage.

Previous research showed that exposure to acute or chronic stress can increase dopamine release in the CNS, and injection of dopamine receptor antagonists into the nucleus accumbens region was found to inhibit the effects of stress (Scornaiencki et al. 2009). Dopamine‐dependent synaptic plasticity in the prefrontal cortex has been found impaired by chronic mild stress, possibly concerning AMPA receptor function (Lamanna et al. 2022). In the present study, the expression of both dopamine D_1_ and especially D_2_ receptor genes was increased by chronic stress, whereas the administration of the *F. subpinnata* extract reduced the expression of these receptors, so it is possible that the extract in part exerted its effects through the reduction of excessive dopamine receptor‐mediated transmission.

Oxidative stress is the result of an imbalance between the formation of ROS and the antioxidant system. Studies have shown that there is a close relationship between oxidative stress and neuronal death. Neuronal damage can indicate an increase in markers of oxidative damage as well as a decrease in antioxidant defense. Chronic stress has many effects on ROS production and nitric oxide production in the brain, which in turn leads to oxidative damage to the CNS. Oxidative stress leads to depression by increasing cytokine levels, decreasing neurogenesis, increasing glutamatergic activity, and inducing apoptosis (Floyd 1999). Plant secondary metabolites have different biochemical and antioxidant effects (Ahmad et al. 2020). These compounds have anti‐inflammatory effects and act as protective agents against oxidative stress. Considering that oxidative stress is relevant to the pathogenesis of anxiety and depression and amnesia (Kobori et al. 2015), it seems that one of the possible mechanisms of reducing anxiety by the *F. subpinnata* extract in stressed animals in this study is the reduction of oxidative stress in the brain tissue.

Overall, in comparison to other extract groups, the 200 mg/kg extract dosage demonstrated superior efficacy in mitigating the effects of chronic unpredictable stress, as indicated by nearly all molecular and behavioral assessments. Remarkably, this dose even enhanced learning and memory performance while reducing anxiety‐like behavior in normal rats (without CUS) across behavioral (measured distance in the Plus Maze test), biochemical (antioxidant assay), and real‐time PCR (with increased *Bdnf*, *TrkB*, *Npy*, *Mr*, *Gr*, and *Gabra 2* expression, and decreased *Grin* and *Gria 1* expression) tests. One significant constraint of this study lies in the absence of experiments conducted on female rats. Consequently, it is advisable to extend the research to explore the impact of this plant specifically on female rat subjects.

## Author Contributions

**Mohammad R. Samandari‐Bahraseman**: investigation, visualization, writing–review and editing, software, writing–original draft. **Keyvan Esmaeilzadeh‐Salestani**: writing–original draft, data curation, software, formal analysis. **Manijeh Dogani**: investigation, writing–original draft, writing–review and editing, software, data curation. **Banafsheh Khaleghdoust**: writing–original draft, formal analysis, methodology. **Nima Hatami**: data curation, software, validation, resources. **Saeed Esmaeili‐Mahani**: methodology, validation, supervision. **Leila Elyasi**: validation, software, data curation. **Evelin Loit**: resources, supervision, writing–review and editing. **Jaanus Harro**: conceptualization, resources, supervision, data curation, formal analysis, project administration, writing–review and editing.

## Conflicts of Interest

The authors declare no conflicts of interest.

### Peer Review

The peer review history for this article is available at https://publons.com/publon/10.1002/brb3.70171.

## Supporting information



Additional supporting information can be found online in the Supporting Information section.

## Data Availability

The study's supporting data can be found in the article, its Supporting Information Materials, and upon request from the corresponding author.
