# Supplementary material for: Antidepressant‐ and Anxiolytic‐Like Effect of the Froriepia subpinnata Extract in the Rat: Neurochemical Correlates
Source: Brain Behav. 2024 Nov 28;14(11):e70171. doi: 10.1002/brb3.70171 (PMC11603432; doi:10.1002/brb3.70171)
Supplement: Supplementary file 1 — Additional supporting information can be found online in the Supporting Information section. [file BRB3-14-e70171-s001.docx]

**Table 1.** Details of materials used in the RT-qPCR procedure.

| **NCBI accession number** | **PCR product size** | **Primer sequence** | **Gene name** |
| --- | --- | --- | --- |
| NM_017008 | 392 | F: GTCTTCACCACCACGGAGAAGGC  R: ATGCCAGTGAGCTTCCCGTTCAGC | ***Gapdh*** |
| XM_008762078 | 343 | F: CGTGATCGAGGAGCTGTTGG  R: CTGCTTCAGTTGGCCTTTCG | ***Bdnf*** |
| XM_039097526.1 | 152 | F: GATCCAGGTCGTGAAGTGGG  R: AGAGGAGTTGGCTGTTCGTG | ***Mr*** |
| NM_003176.3 | 123 | F: ACCCTGCATGTATGACCAATGT  R: TTAGGAACTGAGGAGAGAAGCAGTA | ***Gr*** |
| [NM-_012614.2](https://www.ncbi.nlm.nih.gov/entrez/viewer.fcgi?db=nucleotide&id=395627640) | 159 | F: GTGTTTGGGCATTCTGGCTG  R: AGTGTCTCAGGGCTGGATCT | ***Npy*** |
| [XM-_039080873.1](https://www.ncbi.nlm.nih.gov/entrez/viewer.fcgi?db=nucleotide&id=1958794708) | 112 | ‌ F: GACTCCGCATCCGAAGATATG  R: TAGGGTCCACAGCTTCTACA | ***Cck*** |
| NM_012731 | 245 | F: TGACGCAGTCGCAGATGCTG  R: TTTCCTGTACATGATGCTCTCTGG | ***TrkΒ*** |
| NM_001287423.1 | 147 | F: CTCATCTCTAGCCAGGTCTACG  R: GTCAGAGTAGATGGACATTCGGG | ***NMDA (Grin1)*** |
| NM_031608.2 | 125 | F: GGACAACTCAAGCGTCCAGA  R: CCACACAGTAGCCCTCATAGC | ***AMPA (Gria1)*** |
| [NM_-013165.3](https://www.ncbi.nlm.nih.gov/entrez/viewer.fcgi?db=nucleotide&id=1937369739) | 111 | F: CCAGGTGAACACCCAACAC  R: GAAGTGAGCCTCCTTTCCTATG | ***Cckbr*** |
| XM_006253600.4 | 168 | \|  \| F: TCCTTCAAGAGGGAGACGAAAG \| \| --- \| --- \|   R: CCACACAAACACATCGAAGGTG | ***D1 Receptor*** |
| XM_006242979.4 | 144 | \|  \| F: GTCCTGTCCTTCACCATCTCC \| \| --- \| --- \|   R: GACCAGCAGAGTGACGATGA | ***D2 Receptor*** |
| [NM-_001135779.3](https://www.ncbi.nlm.nih.gov/entrez/viewer.fcgi?db=nucleotide&id=2072749964) | 135 | F: GCTTCTTGTTTGGTTCTGGAGTAG  R: AAGAGAAAGGCTCCGTCATG | ***Gaba-Ra2*** |


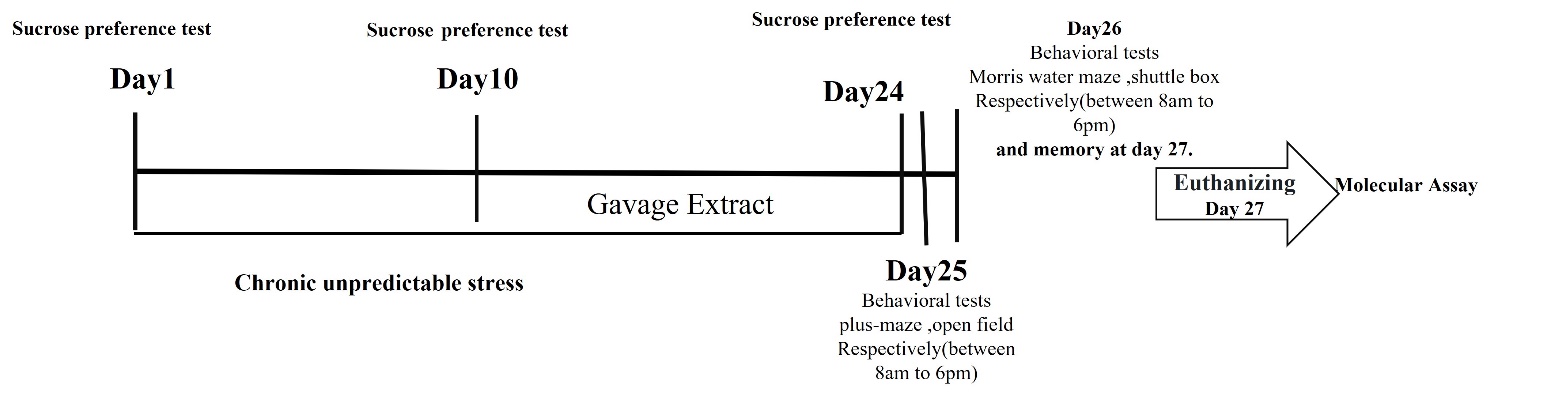


**Figure 1**. The study flow diagram.


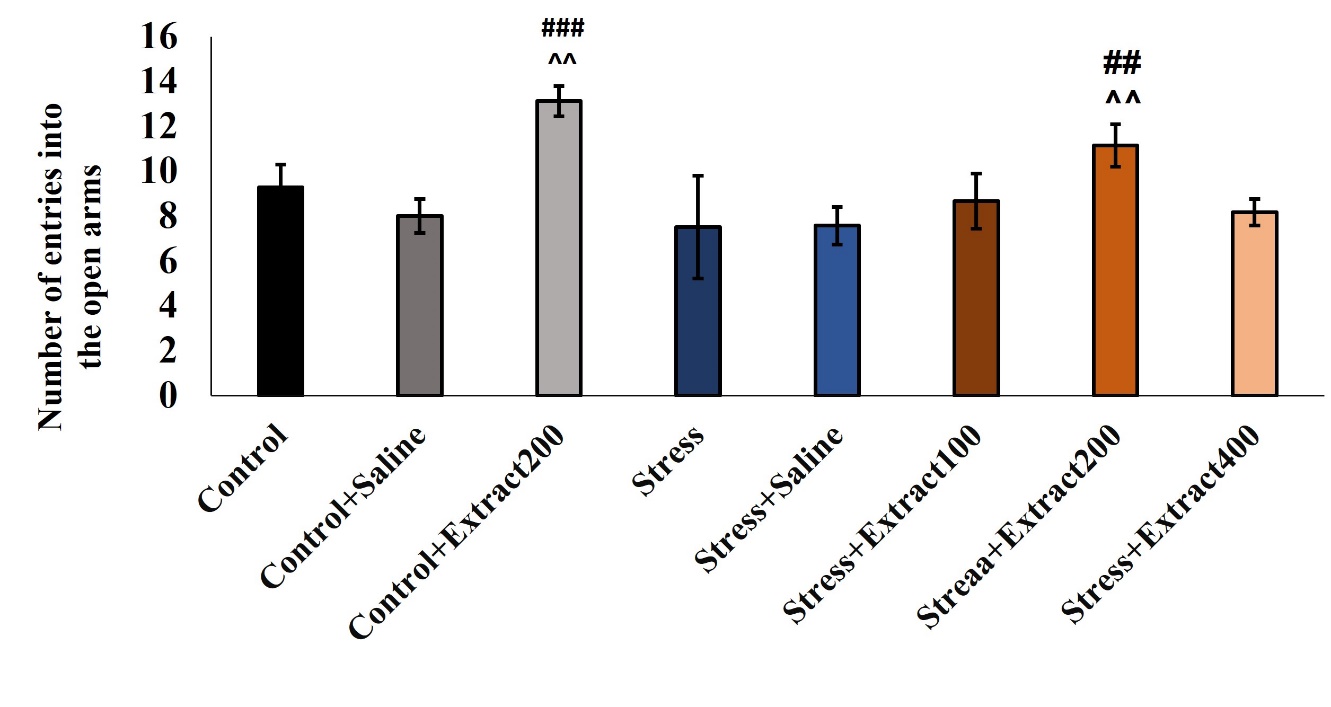


**Figure 2.** Effects of Stress and chronic treatment with extract (100, 200, and 400 mg/kg, po.) on number of entries into the open arms. Each point represents the mean value ± SEM. ^##^P < 0.01 and ^###^P < 0.001 versus Stress group, ^^^^P < 0.01versus Stress+ Extract 100 group. Number of tasted rats for each group = 8.


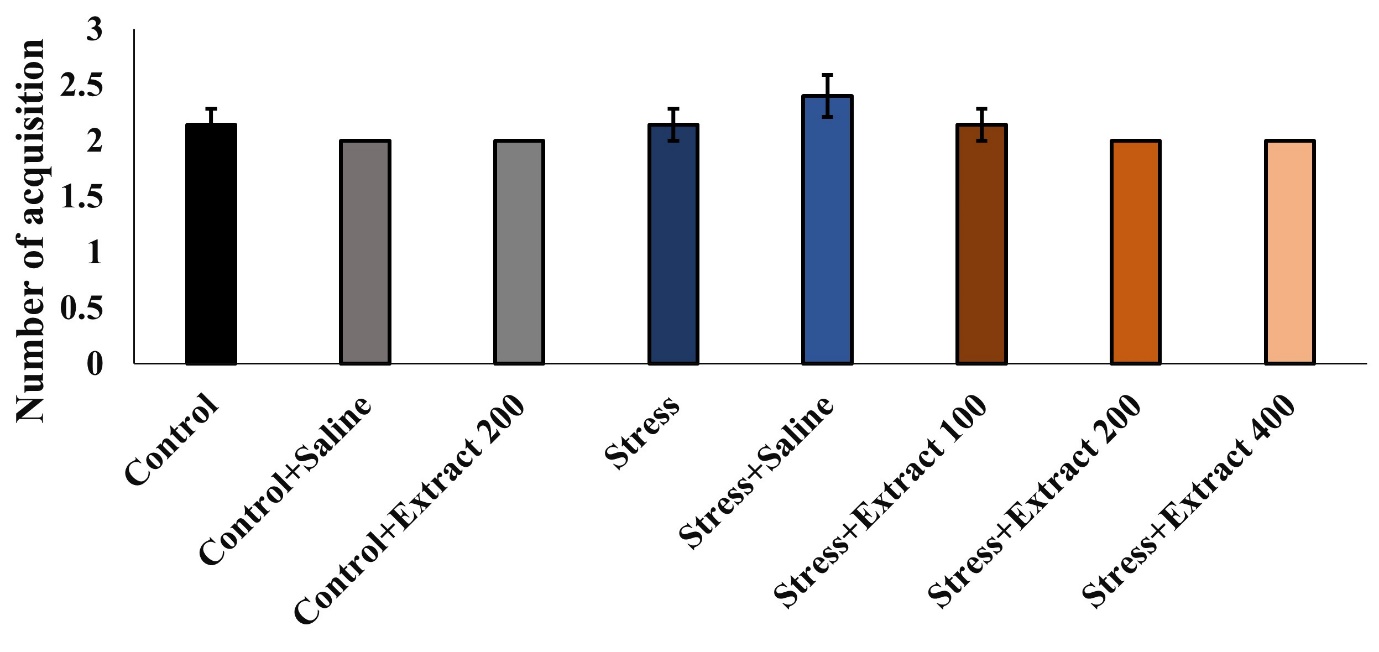


**Figure 3.** Effects of Stress and chronic treatment with extract (100 mg/kg, 200 mg/kg and 400 mg/kg, po.) on number of acquisitions in shuttle box. Each point represents the mean value ± SEM. Number of tasted rats for each group = 8.
